# Effects of Aerobic, Resistance, and Combined Exercise Training on Psychiatric Symptom Severity and Related Health Measures in Adults Living With Schizophrenia: A Systematic Review and Meta-Analysis

**DOI:** 10.3389/fcvm.2021.753117

**Published:** 2022-02-08

**Authors:** Shannon S. D. Bredin, Kai L. Kaufman, Maddison I. Chow, Donna J. Lang, Nana Wu, David D. Kim, Darren E. R. Warburton

**Affiliations:** ^1^Physical Activity Promotion and Chronic Disease Prevention Unit, University of British Columbia, Vancouver, BC, Canada; ^2^Laboratory for Knowledge Mobilization, University of British Columbia, Vancouver, BC, Canada; ^3^Cardiovascular Physiology and Rehabilitation Laboratory, University of British Columbia, Vancouver, BC, Canada; ^4^Department of Radiology, University of British Columbia, Vancouver, BC, Canada; ^5^Department of Anesthesiology, Pharmacology and Therapeutics, University of British Columbia, Vancouver, BC, Canada

**Keywords:** health benefits, schizophrenia, psychiatric symptoms, exercise training, aerobic training, resistance training

## Abstract

Previous research has demonstrated the efficacy, effectiveness, and safety of exercise training in persons living with schizophrenia. However, the optimal exercise training program remains unclear. The aim of this paper was to conduct a systematic review and meta-analysis of the effects of aerobic, resistance, and combined aerobic and resistance training on health-related physical fitness and positive and negative symptoms in persons living with schizophrenia. Six electronic databases were searched systematically from their inception to December 2020 [MEDLINE, Embase, Cochrane Central Register of Controlled Trials (CENTRAL), Web of Science, SPORTDiscus, and Cumulative Index to Nursing and Allied Health Literature (CINAHL)] to identify literature examining the effects of exercise training on psychiatric symptoms and health-related physical fitness indicators in persons living with schizophrenia. A total of 22 studies (*n* = 913) were included in this review, and 12 studies (*n* = 554) included within the meta-analysis reported the effects of exercise training (aerobic, resistance, and combined aerobic and resistance) in persons living with schizophrenia. Aerobic training had a significant decrease on Positive and Negative Syndrome Scale (PANSS) negative scores (ES −2.28, 95% CI −3.57 to −1.00; *p* = 0.0005) and PANSS general scores (ES −2.51, 95% CI −3.47 to −1.55; *p* < 0.00001). Resistance training did not lead to significant effects on PANSS total scores. Combined aerobic and resistance training did not lead to significant changes in body mass index, PANSS positive scores, or PANSS total scores. However, grouping together the results from all exercise training modalities (including aerobic training, resistance training, and combined aerobic and resistance training) revealed significant effects on body mass index (ES 1.86, 95% CI 0.84 to 2.88; *p* = 0.0003), maximal/peak oxygen consumption (ES 2.54, 95% CI 1.47 to 3.62; *p* = < 0.00001), body weight (ES 6.58, 95% CI 2.94 to 10.22; *p* = 0.0004), PANSS negative scores (ES −1.90, 95% CI −2.70 to −1.10; *p* < 0.00001), and Scale for the Assessment of Negative Symptoms (SANS) total (ES −14.90, 95% CI −22.07 to −7.74; *p* < 0.0001). Collectively, these findings support the importance of exercise participation (aerobic and resistance training) in persons living with schizophrenia.

## Introduction

Schizophrenia is a severe mental illness characterized by a combination of psychiatric symptoms, with typical onset occurring in early adulthood. Psychiatric symptoms include positive, negative, and cognitive symptoms, which can have significant effects on daily living, social interactions, and functional capacity in persons living with schizophrenia (1, 2). Positive symptoms can be described as excessive or an exaggeration of normal function, which may include hallucinations, bizarre behaviors, and delusions (1). Negative symptoms can be characterized as a decrease in normal function and typically includes symptoms such as apathy, social withdrawal, and lethargy (1). Furthermore, additional symptoms may be associated with the impairment of cognitive functioning and include behaviors such as a deficit of executive functioning with disorganized speech and short-term memory (1). Schizophrenia affects ~1% of the population worldwide (3), with no significant differences in prevalence rates between males and females (4). In comparison to the general population, schizophrenia can increase the risk of comorbidity and premature mortality (5), reducing lifespan by ~25 years (6, 7). Cardiovascular disease is considered to be the major contributor to the increased risk for premature mortality (5, 8).

Antipsychotic medications are typically considered as a preferred method of treatment for schizophrenia; however, evidence has revealed associated adverse side effects, causing significant concern for long-term health (9, 10). First generation antipsychotics typically have an increased efficacy in treating positive symptoms; yet, they often have reduced efficacy in treating negative symptoms (10). Additionally, first-generation antipsychotic medications can cause side effects such as extrapyramidal symptoms, including dystonia, bradykinesia, and tardive dyskinesia (11). Second-generation antipsychotics were first introduced to primarily increase the efficacy of reducing negative symptoms and incidences of neurological side effects. Therefore, second-generation antipsychotics are commonly utilized to treat psychiatric symptoms in schizophrenia (9, 11, 12). While second-generation antipsychotics have significant benefits for persons living with schizophrenia, the associated side effects must be considered by healthcare professionals (9, 12). Second-generation antipsychotics (such as clozapine) have been associated with increased risks of adverse cardiometabolic effects such as significant weight gain, hyperglycaemia, dyslipidemia, and cardiovascular disease (12–14). Recently, Kim et al. (8) also revealed that the exposure to clozapine was associated with reduced cardiovascular fitness in persons living with chronic schizophrenia.

The increased rates of morbidity among this population are not solely caused by the effects of antipsychotics (12, 13). Persons living with schizophrenia have a higher prevalence of unhealthy lifestyle behavior choices placing them at an increased risk of morbidity and premature mortality (6, 14, 15). For instance, schizophrenia has been associated with a greater prevalence of cigarette smoking, with a reported rate of 90% of individuals engaging in this behavior (14). Furthermore, poor diet and alcohol consumption and substance use is associated with an increased risk of morbidity among this population (6, 14, 16, 17). Additionally, individuals living with schizophrenia have higher rates of sedentary behavior and physical inactivity, with 70–75% of individuals categorized as physically inactive, and a 1.5 to 2 times greater risk of being classified as overweight when compared to the general population (14, 18). A meta-analysis revealed that persons living with schizophrenia exhibit significantly lower maximal/peak oxygen consumption (VO_2_max/peak) in comparison to apparently healthy controls (19).

In consideration of the increased health risks commonly linked to schizophrenia, the use of exercise and/or regular physical activity has been investigated as a potential adjunct therapy (20–22). The role of regular physical activity and exercise participation on reducing the risk for premature all-cause mortality and diverse chronic medical conditions (such as cardiometabolic disease and hypertension) is well established (23). Increasing evidence has shown that regular physical activity and/or exercise participation can improve quality of life, increase functional capacity, improve cardiorespiratory fitness, and increase muscular strength in persons living with major mental illness (21, 24–26). Also, regular physical activity and/or exercise can improve symptom severity, reduce depression, improve cognition, and trigger hippocampal growth (27). In schizophrenia, regular exercise and physical activity participation may also help to counteract some of the side effects associated with antipsychotic medications (14). For instance, psychiatric symptoms, particularly negative symptoms, can be difficult to effectively manage with the use of antipsychotic medication and can greatly influence rates of non-compliance to treatment (14, 20, 21, 28, 29). The engagement in physical activity/exercise can be advantageous in alleviating these symptoms and lead to a reduction in dosage of antipsychotic medications (30, 31). The benefits of physical activity/exercise participation may be even greater in patients with treatment-resistant psychosis (27).

To date, studies have examined various exercise modalities, such as aerobic, resistance, and a combination of aerobic and resistance training as an adjunct treatment for persons living with schizophrenia (21, 32). Exercise interventions have examined various health-related physical fitness indicators [such as body weight, body mass index (BMI), and VO_2_max_/_peak] in response to diverse exercise modalities (15, 20–22, 25, 27, 29, 33–40). However, to the best of our knowledge, no meta-analysis has explored the optimal training exercise program in persons living with schizophrenia. Therefore, this systematic review and meta-analysis was designed to examine the effectiveness of aerobic, resistance, and combined aerobic and resistance training on improving psychiatric symptoms and health-related physical fitness indicators in persons living with schizophrenia.

## Materials and Methods

**Figure 1:**
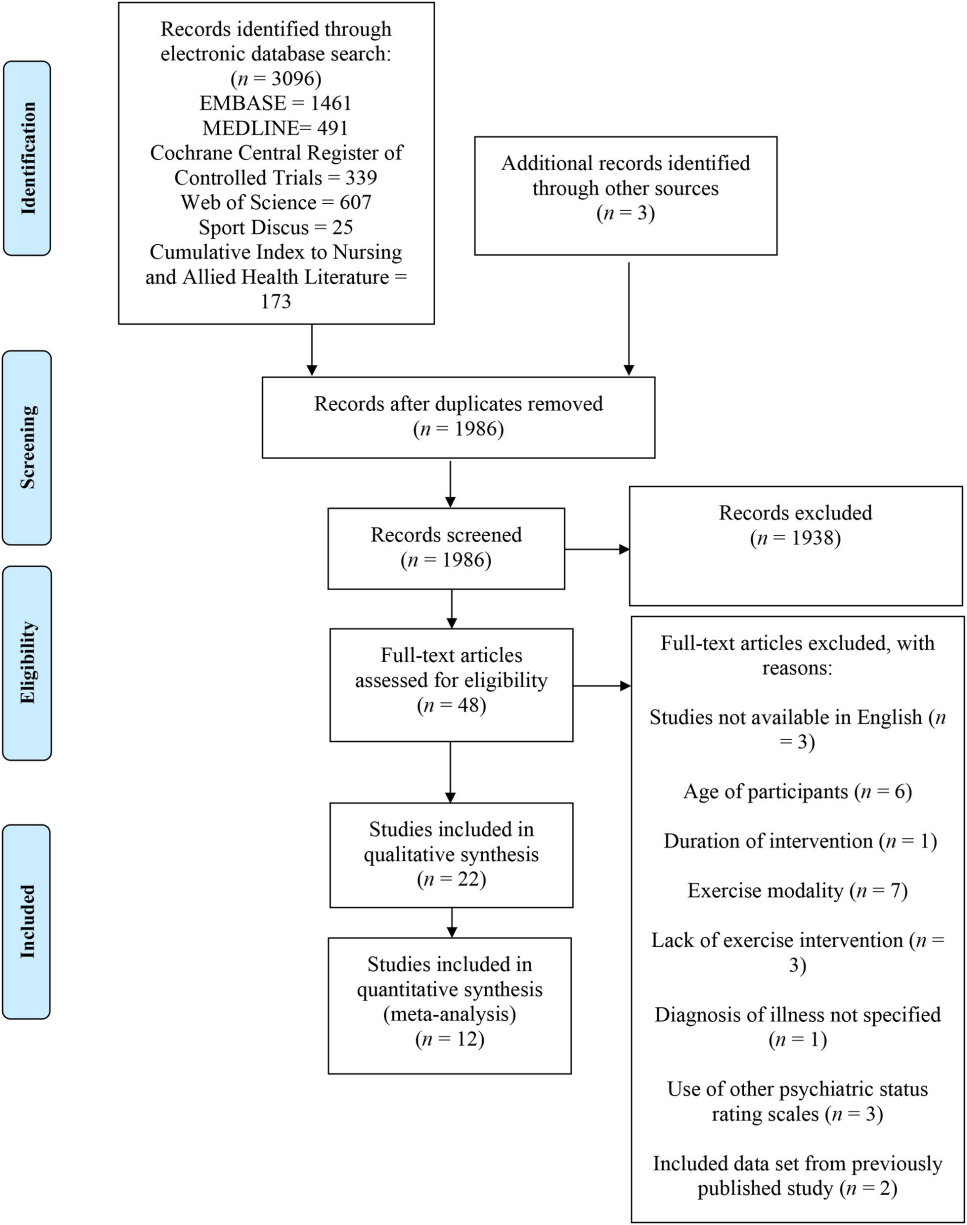
Results of the systematic review.

This systematic review and meta-analysis adheres to the guidelines established by the Preferred Reporting Items for Systematic Reviews and Meta-Analyses (PRISMA) (Figure 1).

### Search Strategy

A preliminary search of the literature was conducted to determine established reviews on the topic of interest prior to performing the systematic review. We conducted the systematic search utilizing the following electronic databases (from their inception to December 2020) to identify relevant studies: EMBASE (Ovid Interface), MEDLINE (Ovid Interface), Cochrane Central Register of Controlled Trials (CENTRAL), Web of Science, SPORTDiscus, and the Cumulative Index to Nursing and Allied Health Literature (CINAHL). Search strategies were tailored for each electronic database. The reviewers conducting the search were not blinded to studies identified. Table 1 provides an example of the literature search strategy and search terms. Additional studies were retrieved through reference lists and through the authors' knowledge.

**Table 1:** Results of the literature search strategy: MEDLINE (Ovid).

**#**	**Search terms**	**Results**
1	exp schizophrenia/ or schizophrenia.mp.	148,490
2	psychosis.mp.	40,821
3	1 or 2	171,975
4	exp exercise/ or exercise.mp	432,398
5	aerobic exercise.mp.	10,439
6	aerobic training.mp.	2,692
7	weight lifting.mp or exp weight lifting/	5,329
8	resistance training.mp. or exp resistance training/	14,795
9	physical activity.mp.	121,398
10	physical exertion.mp. or exp physical exertion/	58,138
11	4 or 5 or 6 or 7 or 8 or 9 or 10	517,276
12	Panss.mp.	4,709
13	BPRS.mp.	2,366
14	Psychiatric Status Rating Scales.mp or exp Psychiatric Status Rating Scales/	85,406
15	Symptom*.mp.	1,243,058
16	(Positive and Negative Syndrome Scale).mp. [mp=title, abstract, original title, name of substance word, subject heading word, keyword heading word, protocol supplementary concept word, rare disease supplementary concept word, unique identifier, synonyms]	4,454
17	Brief Psychiatric Rating Scale.mp. or ex Brief Psychiatric Rating Scale/	3,995
18	12 or 13 or 14 or 15 or 17	1,294,706
19	3 and 11 and 18	491

Exp, explode; mp, title, abstract, original title, name of substance word, subject heading word, keyword heading word, protocol supplementary concept word, rare disease supplementary concept word, unique identifier, synonyms; Asterisk (*), match all terms beginning with a word root.

### Study Selection

Eligible studies for this systematic review met the following inclusion criteria: the study population included individuals between the ages of 18–65 years of either sex, living with a major mental illness, including schizophrenia, first episode psychosis, schizoaffective disorder, schizophreniform disorder, bipolar disorder, and psychotic disorder not otherwise specified. Participants were also required to have received or were currently receiving treatment for their condition and were prescribed antipsychotic medication. Acceptable exercise modalities included aerobic, resistance, or a combination of both aerobic and resistance training with the goal of reducing psychiatric symptoms and improving health-related physical fitness indicators in persons living with schizophrenia. The exercise interventions were required to have been a minimum duration of six weeks in length or longer. The key outcome was to evaluate the positive and negative symptoms in persons living with schizophrenia using valid psychiatric status rating scales, including the Positive and Negative Syndrome Scale (PANSS), Brief Psychiatric Rating Scale (BPRS), Scale for the Assessment of Positive Symptoms (SAPS), and/or Scale for the Assessment of Negative Symptoms (SANS). The secondary outcomes included health-related physical fitness indicators (body weight, BMI, and VO_2_max/peak). There were no limitations placed on study design. Comparisons were made between pre and post scores within each group to determine the effectiveness of the exercise intervention. Furthermore, when feasible, pre and post scores were analyzed between the exercise intervention group and a control group.

We excluded studies that included the following: studies that examined the effectiveness of high intensity interval training and/or mind-body exercises (such as yoga and tai chi) and studies that were not published in English. Studies that utilized symptom scales not identified within the inclusion criteria were also deemed ineligible for this systematic review. Two of the authors (K.L.K. and M.I.C.) independently conducted the search using the six outlined electronic databases and systematically scanned all identified study titles, abstracts, and keywords. In addition, both authors independently examined the full articles of the remaining studies ensuring that all inclusion criteria were met. All excluded studies were removed and the reasons for exclusion were documented. Uncertainties regarding inclusion were resolved by consensus, or by discussion with third, fourth, and fifth reviewers (S.S.D.B., D.E.R.W., and D.D.K.). The review process was guided directly by a professor with expert knowledge of systematic reviews (S.S.D.B.).

### Data Extraction and Quality Assessment

Two reviewers (K.L.K. and M.I.C.) independently extracted the relevant data from the selected articles using a standardized template. Authors were contacted directly to provide clarifications on data that was not included within the publication. Data extracted from the studies included: study purpose, study design, number of participants (*N*), mean age ± standard deviation and age range, sex distribution, diagnosis, mean time since diagnosis, medications, intervention, program duration, baseline health measures, outcome health measures, within group differences, between group differences, symptom severity, outcome symptom severity, within group differences, between group differences, limitations, adherence rates to program, rates of dropout, reasons for dropout, hospitalization duration, location of participant recruitment, and take home message/recommendations. Reviewers were not blinded to publication authors or journals during the extraction process.

Using the Downs and Black Quality Index (41), two of the authors (K.L.K. and M.I.C.) independently assessed the methodological quality of the included studies in alignment with the items established in the valid and reliable tool. Any discrepancies were assessed by another reviewer (N.W.). The checklist evaluation includes a total of 27 questions, with a maximum score of 32, assessing the following sub-scales: (i) reporting, (ii) external validity, (iii) internal validity, bias, (iv) internal validity, confounding, and (v) power. The maximal score signifies the highest level of methodological quality.

### Data Synthesis and Analysis

A meta-analysis was performed utilizing the Review Manager program (RevMan version 5.1, Cochrane Collaboration) to synthesize and analyze the extracted data from the included studies. Results of the meta-analysis were reported as mean effect size and 95% confidence interval (CI) and were displayed using forest plot figures. The degree of heterogeneity across the studies was quantified using the I-squared test and chi-squared test, with 95% CI. Fixed effect models were utilized to illustrate data with lower degrees of heterogeneity (<50%), whereas random effects models were chosen when a significant degree of heterogeneity was present. When feasible, sensitivity analyses were completed by sequentially eliminating individual studies to determine if the results were greatly influenced by a particular study. When appropriate, subgroup analyses were examined between exercise intervention duration, frequency, and intensity. An assessment of publication bias was conducted for this meta-analysis using funnel plots. All studies included in the meta-analysis included an intervention and control group, with some of the control groups engaging in an additional form of exercise.

## Results

### Study Selections and Characteristics

A search of six electronic databases was conducted for this systematic review. Additional studies were included based on the authors' knowledge. After removing duplicates, titles, abstracts, and keywords were screened to identify potential studies. Full articles were then reviewed to determine if eligibility criteria were met (Figure 1). A total of 26 studies were excluded with reasons from this systematic review. In total, 22 studies published from 2005 (33) to 2020 (42) were deemed eligible through our inclusion criteria and examined the effects of exercise training for persons living with schizophrenia. The included studies consisted of aerobic training (*n* = 12), resistance training (*n* = 3), and combined aerobic and resistance training (*n* = 7). The results of the Downs and Black Quality Index assessment were consistent with good quality scores for the included studies (Table 2). The mean score was 24.8 out of 32, with a range of scores between 18 and 29. A meta-analysis was conducted using 12 of the included studies (20, 21, 25, 29, 34, 37–39, 42–44, 48). The summary of the quantitative results by modality of exercise and outcome are outlined in Table 3. Funnel plots were used to assess the publication bias for each outcome within the meta-analysis. It was determined that all figures were relatively symmetrical around the effect size, indicating that there was no publication bias present within the meta-analysis. Studies not included in the meta-analysis were excluded for the following reasons: lack of control group (*n* = 5) (15, 22, 27, 35, 40), missing data (*n* = 3) (33, 45, 46), and insufficient data to conduct a meta-analysis (*n* = 2) (36, 47).

**Table 2:** Downs and black quality index assessment for methodological quality.

**References**	**Reporting (/11)**	**External validity (/3)**	**Bias (/7)**	**Confounding (/6)**	**Power (/5)**	**Total (/32)**
Acil et al. (43)	7	2	4	4	5	22
Beebe et al. (33)	8	1	5	5	2	21
Bredin et al. (22)	9	2	5	5	3	24
Browne et al. (15)	6	1	4	2	5	18
Curcic et al. (34)	8	2	4	4	5	23
Dodd et al. (35)	9	2	4	3	4	22
Firth et al. (29)	8	3	4	3	4	22
Heggelund et al. (25)	10	1	4	4	3	22
Kern et al. (36)	10	2	7	5	5	29
Korman et al. (40)	10	3	5	3	5	26
Loh et al. (44)	10	2	6	5	5	28
Malchow et al. (45)	9	2	5	4	5	25
Maurus et al. (38)	11	2	5	5	2	25
Pajonk et al. (46)	9	3	6	6	4	28
Ryu et al. (47)	9	2	5	5	5	26
Scheewe et al. (20)	11	2	5	5	5	28
Senormanci et al. (42)	9	3	4	3	5	24
Shimada et al. (48)	9	1	5	6	5	26
Silva et al. (21)	9	2	6	5	5	27
Su et al. (37)	10	2	5	5	5	27
Svatkova et al. (39)	10	2	6	4	5	27
Woodward et al. (27)	10	2	4	6	4	26

**Table 3:** Summary of quantitative results by modality of exercise and outcome.

**Modality of exercise**	**Examined outcome**	**Number of studies**	**Results**
Aerobic Training	PANSS Positive	4	Slightly favors exercise group Not statistically significant
Aerobic Training	PANSS Negative	4	Favors exercise group Statistically significant
Aerobic Training	PANSS General	3	Favors exercise group Statistically significant
Resistance Training	PANSS Total	3	Slightly favors exercise group Not statistically significant
Combined (Aerobic and Resistance Training)	Body Mass Index	3	Favors control group Statistically significant
Combined (Aerobic and Resistance Training)	PANSS Positive	3	Slightly favors exercise group Not statistically significant
Combined (Aerobic and Resistance Training)	PANSS Total	4	Slightly favors control group Not statistically significant
All Exercise Training Modalities	Body Mass Index	4 (5 trials)	Favors control group Statistically significant
All Exercise Training Modalities	Maximal Oxygen Consumption	4	Favors exercise group Statistically significant
All Exercise Training Modalities	Body Weight	2 (3 trials)	Favors control group Statistically significant
All Exercise Training Modalities	PANSS Positive	8 (9 trials)	Favors exercise group Not statistically significant
All Exercise Training Modalities	PANSS Negative	8 (9 trials)	Favors exercise group Statistically significant
All Exercise Training Modalities	PANSS General	6 (7 trials)	Favors exercise group Not statistically significant
All Exercise Training Modalities	PANSS Total	8 (9 trials)	Favors exercise group Not statistically significant
All Exercise Training Modalities	SANS	3	Favors exercise group Statistically significant

Participant characteristics, interventions, baseline and outcome health measures, baseline and outcome psychiatric symptom severity scores, and intervention adherence rates are outlined in Supplementary Tables 1–3. The exercise interventions of the included studies investigated different exercise modalities (aerobic, resistance, or combined aerobic and resistance training), program durations, exercise intensities, and exercise frequencies. An included study implemented both a resistance exercise group and a combined exercise group in comparison to a control group and therefore, data for this study was reported as two independent trials. To evaluate symptom severity scores, 17 of the included studies utilized the PANSS. Of these studies, 11 reported PANSS positive scores, 11 reported PANSS negative scores, and seven reported PANSS general scores; while 14 reported PANSS total scores. Additionally, three studies used the BPRS, and four studies utilized the SANS and/or the SAPS. In addition, many of the included studies examined the effectiveness of various exercise modalities on improving health-related physical fitness measures, such as body mass index (*n* = 11), VO_2_max/peak (*n* = 9), and body weight (*n* = 6).

### Participants and Exercise Intervention

A combined total of 814 participants living with severe mental illness and 83 healthy controls were included in this systematic review. The participant sample size ranged from 8 to 104, with participants' diagnoses including schizophrenia, schizoaffective disorder, schizophreniform disorder, bipolar disorder, first episode psychosis, or psychotic disorder not otherwise specified. All participants (excluding healthy controls) were currently receiving antipsychotic medication as treatment. It is important to note that two studies included participants living with treatment-resistant schizophrenia (27, 35). In total, the exercise intervention duration ranged between 8 and 26 weeks in length, and incorporated between one and five sessions per week, with the majority including three exercise sessions per week (11 studies) or twice per week (eight studies). In addition, the exercise sessions ranged between 30 and 60 min. Exercise intensity was reported in terms of percentage of maximal aerobic power (VO_2_max) or VO_2_peak, maximum heart rate, heart rate reserve, rating of perceived exertion, talk test, or percentage of 1-repetition maximum. The intensity of exercise ranged between 40 and 80% VO_2_max or VO_2_peak, 55 and 75% maximum heart rate, 30 and 75% heart rate reserve, and 60 and 85% 1 repetition maximum. Exercise equipment for aerobic and resistance training included treadmill, cycle ergometer, elliptical, recumbent bike, rowing machine, cross trainer, resistance bands, dumbbells, and various small training equipment.

### Aerobic Training

**Figure 2:**
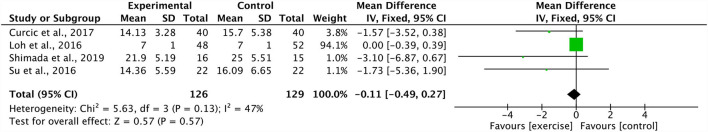
The effects of aerobic training on PANSS positive scores.

**Figure 3:**
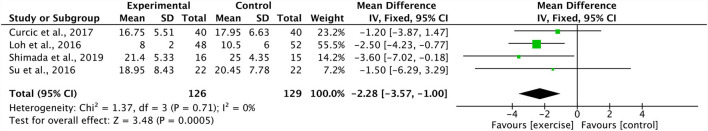
The effects of aerobic training on PANSS negative scores.

**Figure 4:**
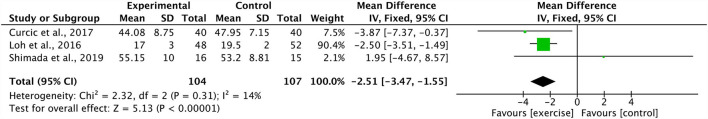
The effects of aerobic training on PANSS general scores.

Four studies combined in a meta-analysis found no significant effect of aerobic training on PANSS positive scores (Effect Size, ES −0.11, 95% CI −0.49 to 0.27; *p* = 0.57) (34, 37, 44, 48). Across these studies, there was a moderate degree of heterogeneity (*I*^2^: 47%; *p* = 0.13) (Figure 2). The sensitivity analysis greatly influenced the results, yielding a significant mean effect (ES −1.86, 95% CI −3.43 to −0.30; *p* = 0.02) with a low degree of heterogeneity (*I*^2^: 0%; *p* = 0.78) (44). Additionally, these four studies were further analyzed in a meta-analysis which identified a significant decrease in PANSS negative scores within the aerobic training group (ES −2.28, 95% CI −3.57 to −1.00; *p* = 0.0005) (34, 37, 44, 48). The degree of heterogeneity was low for these studies (*I*^2^: 0%; *p* = 0.71) (Figure 3). Further, three studies were pooled, revealing a significant reduction on effect of PANSS general scores, favoring the aerobic training group (ES −2.51, 95% CI −3.47 to −1.55; *p* < 0.00001) (34, 44, 48) (Figure 4). A low degree of heterogeneity was detected across the studies (*I*^2^: 14%; *p* = 0.31). Due to insufficient study data, we were unable to conduct a meta-analysis on VO_2_max/peak; however, there is growing evidence from randomized trials (49) to support the potential for significant changes in aerobic capacity after exercise interventions in persons living with schizophrenia.

### Resistance Training

**Figure 5:**
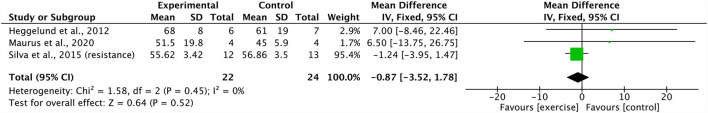
The effects of resistance training on PANSS total scores.

Three studies were included in a meta-analysis revealing no significant effect of resistance training on PANSS total scores (ES −0.87, 95% CI −3.52 to 1.78; *p* = 0.52) (21, 25, 38). The degree of heterogeneity was low (*I*^2^: 0%; *p* = 0.45) (Figure 5). A sensitivity analysis revealed a shift in the results, changing the mean effect size to positive, however, it did not change the overall significance of the effect (21).

### Combined Aerobic and Resistance Training

**Figure 6:**
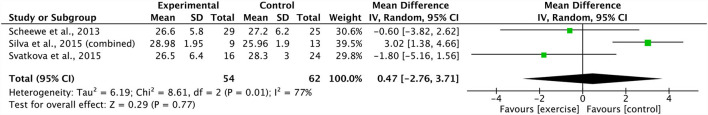
The effects of combined aerobic and resistance training on body mass index scores.

**Figure 7:**
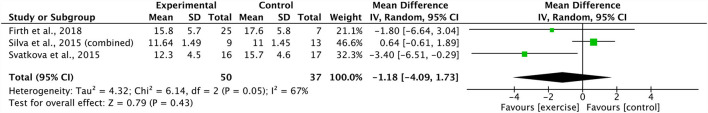
The effects of combined aerobic and resistance training on PANSS positive scores.

**Figure 8:**
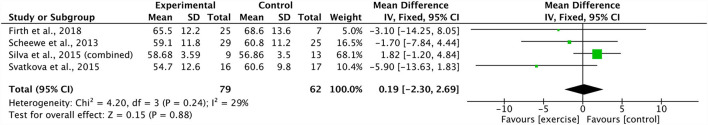
The effects of combined aerobic and resistance training on PANSS total scores.

A meta-analysis pooling three studies identified no significant effect of combined aerobic and resistance training on body mass index (ES 0.47, 95% CI −2.76 to 3.71; *p* = 0.77) (20, 21, 39). There was a substantial degree of heterogeneity across these studies (*I*^2^: 77%; *p* = 0.01) (Figure 6). Sensitivity analysis revealed shifts in the results; however, there was no effect on the overall significance. In addition, three studies were grouped, revealing a non-significant effect of combined aerobic and resistance training on PANSS positive scores (ES −1.18 95% CI −4.09 to 1.73; *p* = 0.43) (21, 29, 39). The degree of heterogeneity across the studies was substantial (*I*^2^: 67%; *p* = 0.05) (Figure 7). The sensitivity analysis revealed significant shifts and changed the overall significance of the results (ES −2.93, 95% CI −5.55 to −0.32; *p* = 0.03) (21). The degree of heterogeneity was low (*I*^2^: 0%; *p* = 0.59). Finally, four studies were pooled, illustrating a non-significant effect of combined aerobic and resistance training on PANSS total scores (ES 0.19, 95% CI −2.30 to 2.69; *p* = 0.88) (20, 21, 29, 39). Heterogeneity was low within these studies (*I*^2^: 29%; *p* = 0.24) (Figure 8). Conducting a sensitivity analysis shifted the mean effect to be negative, but did not affect the overall significance (21).

### All Exercise Training Modalities Included

Four studies (five trials) were pooled in a meta-analysis displaying a significant effect of all exercise training modalities (aerobic, resistance, and combined training) on body mass index (ES 1.86, 95% CI 0.84 to 2.88; *p* = 0.0003) (20, 21, 38, 39). There was a moderate degree of heterogeneity across the trials (*I*^2^: 42%; *p* = 0.14). In addition, four studies were combined, showing a significant increase in VO_2_max/peak of all exercise training modalities (ES 2.54, 95% CI 1.47 to 3.62; *p* < 0.00001), favoring the exercise group (20, 34, 37, 39). The heterogeneity of the combined studies was low (*I*^2^: 0%; *p* = 0.78). Two studies (three trials) were combined revealing a significant effect of all exercise training modalities on body weight (ES 6.58, 95% CI 2.94 to 10.22; *p* = 0.0004) (21, 25). These combined trials showed a low degree of heterogeneity (*I*^2^: 0%; *p* = 0.40). Furthermore, eight studies (nine trials) were pooled, yielding a non-significant effect on PANSS positive scores for all exercise training modalities (ES −0.81, 95% CI −1.74 to 0.13; *p* = 0.09) (21, 29, 34, 37–39, 44, 48). A moderate degree of heterogeneity was identified within these trials (*I*^2^: 57%; *p* = 0.02). A sensitivity analysis slightly influenced the results, indicating a significant effect on PANSS positive scores (ES −1.20, 95% CI −2.32 to −0.08; *p* = 0.04) with a moderate degree of heterogeneity (*I*^2^: 58%; *p* = 0.02) (44). The subgroup differences between intervention duration (>12 weeks and ≤ 12 weeks) and frequency (<3 sessions per week and ≥3 sessions per week) within all exercise training modalities (aerobic, resistance, and combined training) on PANSS positive scores, were not statistically significant (*p* = 0.16; *p* = 0.80). In addition, using the same eight studies (nine trials), all exercise training modalities had a significant effect on PANSS negative scores (ES −1.90, 95% CI −2.70 to −1.10; *p* < 0.00001) (21, 29, 34, 37–39, 44, 48). There was low heterogeneity among these studies (*I*^2^: 18%; *p* = 0.28). The subgroup differences between intervention duration (>12 weeks and ≤ 12 weeks), frequency (<3 sessions per week and ≥3 sessions per week), and intensity (moderate; moderate-vigorous) within all exercise training modalities (aerobic, resistance, and combined training) on PANSS negative scores, were not statistically significant (*p* = 0.16; *p* = 0.09; *p* = 0.11). A total of six studies (seven trials) were included in the meta-analysis demonstrating no significant effect of all exercise training modalities on PANSS general scores (ES −0.02, 95% CI −2.50 to 2.45; *p* = 0.99) (21, 29, 34, 38, 44, 48). In addition, there was a considerable degree of heterogeneity across these studies (*I*^2^: 87%; *p* = < 0.00001). Furthermore, a sensitivity analysis had little effect on results and did not impact the significance of this effect. Eight studies (nine trials) were pooled in a meta-analysis demonstrating a non-significant effect of all exercise training modalities on PANSS total scores (ES −2.01, 95% CI −5.13 to 1.10; *p* = 0.21) (20, 21, 25, 29, 34, 38, 39, 48). The degree of heterogeneity across these studies was moderate (*I*^2^: 54%; *p* = 0.02). Sensitivity analysis showed a considerable shift, yielding a significant mean effect (ES −2.73, 95% CI −4.74 to −0.71; *p* = 0.008) (21). These trials showed a low degree of heterogeneity (*I*^2^: 35%; *p* = 0.15). The subgroup differences between intervention duration (>12 weeks and ≤ 12 weeks), frequency (<3 sessions per week and ≥3 sessions per week), and intensity (moderate; moderate-vigorous) within all exercise training modalities (aerobic, resistance, and combined training) on PANSS total scores, were not statistically significant (*p* = 0.41; *p* = 0.29; *p* = 0.10). Finally, three studies were grouped in a meta-analysis and displayed a significant decrease in SANS total scores in all exercise training modalities (ES −14.90, 95% CI −22.07 to −7.74; *p* < 0.0001). The heterogeneity across these studies was low (*I*^2^: 0%; *p* = 0.46).

## Discussion

This systematic review consisted of 22 studies, with a total of 12 studies included in the meta-analysis to examine the effectiveness of aerobic, resistance, and combined aerobic and resistance training on improving psychiatric symptoms and health-related physical fitness indicators in persons living with schizophrenia. The findings from our meta-analysis reveal significant effects of exercise training on psychiatric symptoms and health-related physical fitness measures (such as body weight, BMI, and VO_2_max/peak). These findings further support the importance of exercise training as an adjunct therapy for persons living with schizophrenia.

### Aerobic Training

An increasing number of studies have investigated the effects of aerobic training on the improvement of health measures and the reduction of psychiatric symptoms in persons living with schizophrenia. Walking has been identified as a preferred method of treatment in individuals living with schizophrenia due to high accessibility and increased levels of motivation (15, 50). In our meta-analysis, four studies included walking/brisk walking demonstrating reductions in PANSS positive, negative, and total scores (34, 44, 48), or BPRS positive and negative scores (36). Exercise duration ranged from 90 to 180 min per week, with two-to-four sessions per week, implemented over a duration of 12 weeks. Two studies began the exercise intervention with relatively short exercise durations and gradually increased to 150 min per week (36, 44). However, Loh et al. (44) acknowledged that participants in the walking group reported greater levels of motivation due to the increased amount of social support provided. Thus, while the results from these moderate-intensity walking studies have demonstrated a relative decrease in positive and negative symptoms, more research is warranted to demonstrate a significant change in symptoms. Similarly, these findings were supported by a recent review (51) suggesting that while the recommendations for optimal aerobic training dosage is still unclear, a duration of 30–40 min, three days per week, over 10–12 weeks resulted in increased health benefits. Importantly, this dosage is well below international physical activity guideline recommendations for apparently healthy individuals, but consistent with other clinical populations that have exhibited low aerobic capacities and high levels of physical inactivity and sedentary behavior (52, 53).

A recent systematic review and meta-analysis revealed significant improvements in both PANSS positive and negative scores for individuals living with schizophrenia within the aerobic exercise intervention group (54). Similarly, our systematic review revealed that aerobic training had a significant influence on PANSS negative scores; however, we did not find a significant change in PANSS positive scores. Due to the limited studies included within our current paper, these findings should be interpreted with caution. There was a moderate degree of heterogeneity across the studies within the PANSS positive scores; however, low heterogeneity was found among these studies for PANSS negative scores. The aerobic studies included in the systematic review by Sabe and colleagues also revealed improvements in VO_2_max/peak scores, suggesting a possible relationship between aerobic capacity and decreased PANSS negative scores (54). In our current work, two included studies demonstrated improvements in VO_2_max/peak (34, 37). Due to the lack of studies, we were not able to conduct a meta-analysis on this outcome. However, there is growing evidence from randomized trials to support the potential for significant changes in aerobic capacity after exercise interventions in persons living with schizophrenia (34, 49). Furthermore, other studies (not employing control groups) have also demonstrated significant (and clinically relevant) improvements in VO_2_max/peak and other markers of aerobic fitness and exercise tolerance (22, 35). This is further supported by a clinical overview and meta-analysis by Vancampfort et al. (19) that reported lower cardiorespiratory fitness levels in persons living with schizophrenia in comparison to apparently healthy controls. The authors provided recommendations for aerobic training to help optimize health benefits (19). These recommendations outlined the importance of administering a risk stratification, assessments for cardiorespiratory fitness, attainable exercise interventions based on fitness level, and support strategies to ensure adherence to programs (19). Further, it is important for clinicians to consider psychiatric symptoms (specifically negative symptoms), elevated BMI scores, reported presence of pain, and comorbidities as factors that can influence the ability to perform and adhere to aerobic training and thus increase health benefits (19). This supports the earlier recommendations of our team (22).

Current literature suggests that aerobic exercise has a significant effect on PANSS general scores by reducing associated symptoms such as depression and anxiety (34, 55). These results are consistent with our findings in this systematic review. Further, findings by Pelham et al. (55) indicates that there is a negative correlation associated between aerobic fitness levels and rates of depression. This suggests that increasing levels of aerobic fitness results in lower levels of depression, leading to lower levels of PANSS general scores (55). This is supported by the findings of Woodward and colleagues that demonstrate a 12-week aerobic or weight-bearing exercise program can improve symptom severity, reduce depression, improve cognition, and trigger hippocampal growth (27).

### Resistance Training

Despite the clear health benefits of engaging in exercise that taxes the musculoskeletal system (56, 57), few studies have examined the effect(s) of resistance training on psychiatric symptoms. Therefore, there is a paucity of evidence surrounding the benefits of resistance training in persons living with schizophrenia (21, 25). The meta-analysis revealed no significant findings with respect to PANSS total scores. Although it has been suggested that resistance training may have a beneficial effect on improving psychiatric symptoms in patients living with schizophrenia (21), a pilot study by Heggelund et al. (25) reported an increase in PANSS total scores following a resistance training intervention. These authors suggested that the findings may be attributed to the natural course of the illness commonly seen in persons living with schizophrenia. This is an important point to consider when comparisons are made to changes seen in other groups (e.g., apparently healthy controls and other clinical populations). Additionally, the authors acknowledged that an 8-week intervention may be too short in duration for participants to adapt to the exercise intervention to produce greater reductions in symptom severity. Moreover, a study by Silva et al. (21) reported significant improvements in PANSS positive, negative, and total scores following a 20-week resistance training intervention.

Although there was insufficient data to conduct a meta-analysis, two studies investigated BMI scores in response to resistance training, revealing equivocal findings. Specifically, Silva et al. (21) revealed minor reductions in BMI scores following a 20-week intervention. In comparison, following a 12-week resistance training intervention, Maurus et al. (38) documented a slight increase in BMI scores. These divergent findings are not unanticipated owing to differences in exercise training programs, treatment patterns, and clinical characteristics of persons living with schizophrenia.

Resistance training reduces all-cause mortality by increasing musculoskeletal fitness and functional independence in the general population (56–58). Therefore, considering the increased risk of secondary complications (such as cardiometabolic disease) and premature mortality, further investigation is warranted to fully examine the effects of resistance training on psychiatric symptoms and health outcomes in schizophrenia (18, 25, 59).

Although further research is clearly warranted, the findings from this systematic review support the implementation of resistance training in persons living with schizophrenia. The optimal dosage, intensity, and type of resistance training remains to be determined. However, there is preliminary evidence supporting two-to-three sessions of resistance training carried out over a period of 12 weeks or more. This is similar to what is seen with the rehabilitation of other chronic medical conditions with low baseline fitness (e.g., chronic heart failure) (60–62).

### Combined Aerobic and Resistance Training

Combined aerobic and resistance training has been shown to decrease cardiovascular disease risk factors and increase VO_2_max in overweight and obese populations when compared to aerobic or resistance training interventions (63, 64). Evidence has revealed that incorporating resistance training into an aerobic exercise intervention may act as a form of therapeutic treatment, as individuals living with schizophrenia receive the benefits of both aerobic and resistance training. In addition, findings suggest that combined training may help to improve body composition and lean body mass in persons living with schizophrenia (21, 26). All participants across three studies included in the meta-analysis reported BMI scores that were categorized as overweight (20, 21, 39). Although the mean effect size was not significant, there was a significant degree of heterogeneity that should be considered when interpreting these findings (Figure 5). It is important to note that a contributing study consisted of a small sample size of solely male participants (21), which may not be an accurate representation of the general clinical population. Considerable limitations such as small sample sizes (21), low compliance rates, high drop-out rates, and symptom severity (20) may be contributing factors to these findings and the associated heterogeneity.

A systematic review by Martin et al., (64) determined that an exercise program involving both aerobic and resistance training led to improvements in PANSS negative and total scores. An additional systematic review by Firth et al. (65) found improvements in psychiatric symptoms (PANSS positive, negative, and total scores) that were only detected through the implementation of programs that included aerobic training. Our systematic review had preliminary evidence to support this conclusion; however, our meta-analysis did not reveal a statistically significant benefit for PANSS total scores. Conducting a sensitivity analysis showed that removing the only study with a positive ES (21) shifted the findings to favor combined training on PANSS total scores. However, these changes did not lead to significant findings. Furthermore, positive symptoms were favored within the combined training groups; however, sensitivity analysis was required to remove the only positive ES in order establish statistical significance in these findings. It is also important to note that the degree of heterogeneity in the meta-analysis was statistically significant across the studies assessing PANSS positive scores; therefore, these findings should be interpreted with caution. There is preliminary evidence to support the efficacy of combined training on psychiatric symptoms. Additional studies are clearly warranted to further investigate the effectiveness of combined training on improving psychiatric symptoms and health outcomes in schizophrenia.

### Limitations

We aimed to evaluate the optimal exercise training program for improving health-related physical fitness and other health indicators in persons living with schizophrenia. However, we acknowledge certain limitations within this systematic review and meta-analysis. First, only 12 out of the 22 included studies were randomized controlled trials or quasi-experimental designs, with a large majority of the remaining articles being pilot or feasibility studies. Further, our findings reflected studies that included relatively small sample sizes, making it challenging to ensure the included sample population was an accurate representation of the clinical population of interest. While some studies reported specific levels of antipsychotic medications, the potential confounding influence of antipsychotic medications were not considered in this review. In addition, not all authors responded to requests made for further information or additional data.

Data from studies that did not implement a control group were not included within the meta-analysis. Therefore, comparisons between the intervention of interest and a control were analyzed. However, many of the control groups within the included studies participated in an alternative form of physical activity. As a result, it is difficult to determine whether the reported changes in health measures and psychiatric symptom scores are a result of the implemented exercise intervention. Due to the limited number of studies selected within this systematic review, there was minimal data available to interpret many of the outcomes. This created a challenge when independently conducting meta-analyses within each exercise modality. To support a greater number of outcomes, meta-analyses were conducted combining all exercise training modalities. Additionally, there were only two studies that included persons with treatment-resistant schizophrenia; however, because these studies did not include a control group, a meta-analysis could not be performed. Increasingly, control groups are not being employed owing to the overwhelming health benefits seen with exercise training in persons living with schizophrenia. In addition, due to the limited number of studies included within the meta-analysis, it was not feasible to conduct subgroup analyses for duration, frequency, and intensity within each exercise modality group (aerobic, resistance, and combined aerobic and resistance training). Therefore, subgroup analyses were only conducted for all exercise training modalities for PANSS positive, PANSS negative, and PANSS total. Finally, it should be noted that the degree of heterogeneity within this meta-analysis ranges from low to considerable, with the majority being low.

### Future Directions

Future studies should carefully examine the risks and adherence rates between modalities of exercise training to outline evidence-based recommendations for exercise training interventions that consider patient interests (such as motivational and support strategies to maximize health benefits associated with exercise training). Moreover, while varying degrees of symptom severities were taken into consideration for this systematic review, further investigation is needed to determine which modality of exercise training is optimal for individuals who may experience severe ratings of psychiatric symptoms and/or may be treatment resistant. Finally, additional studies are warranted to clearly examine the ideal dosages (frequency, intensity, type, and time) of aerobic, resistance, and combined training to achieve optimal health benefits in persons living with schizophrenia. This research is important in the context of recent findings that demonstrate an exacerbation of symptoms following exercise training in individuals living with schizophrenia receiving certain antipsychotic treatment regimens (66).

## Conclusion

Our findings suggest that different modalities of exercise may play an important role in reducing psychiatric symptoms and improving health-related physical fitness outcomes in persons living with schizophrenia. These findings are directly aligned with previous research that highlights the importance of incorporating exercise as an adjunct treatment to improve health and wellbeing in this population (30, 65). More studies are needed to determine which type of exercise has increased efficacy in reducing psychiatric symptoms and various health measures. There is growing evidence that 90 min of weekly moderate-to-vigorous intensity exercise aerobic or combined aerobic and resistance exercise can lead to a significant reduction in symptoms in persons living with schizophrenia, with the potential for health benefits at relatively low volumes and intensities of exercise.

## Data Availability Statement

The original contributions presented in the study are included in the article/Supplementary Material, further inquiries can be directed to the corresponding author/s.

## Author Contributions

SSDB, KLK, MIC, DJL, and DERW: conceptualization. SSDB, KLK, MIC, DJL, NW, DDK, and DERW: methodology, writing—review, and editing. SSDB, KLK, MIC, and DERW: formal analysis, investigation, and writing—original draft preparation. SSDB, KLK, MIC, NW, and DERW: data curation. SSDB and DERW: supervision, project administration, funding acquisition, and resources. All authors contributed to the article and approved the submitted version.

## Funding

SSDB was supported by the Canadian Institutes of Health Research, MITACs and the Social Sciences and Humanities Research Council of Canada. DERW was funded by the Canadian Institutes of Health Research, MITACs, the Social Sciences and Humanities Research Council, the NIB Trust Fund, and the Natural Sciences and Engineering Research Council of Canada and supported by a CIHR New Investigator Award and a Michael Smith Foundation for Health Research Clinical Scholar Award. DJL has received operating funds from the Canadian Institutes for Health Research, the BC Mind Foundation, and the Provincial Health Services Authority of BC in conjunction with the B.C. Mental Health and Addictions Research Institute.

## Conflict of Interest

The authors declare that the research was conducted in the absence of any commercial or financial relationships that could be construed as a potential conflict of interest.

## Publisher's Note

All claims expressed in this article are solely those of the authors and do not necessarily represent those of their affiliated organizations, or those of the publisher, the editors and the reviewers. Any product that may be evaluated in this article, or claim that may be made by its manufacturer, is not guaranteed or endorsed by the publisher.
